# Synthetic Associations Created by Rare Variants Do Not Explain Most GWAS Results

**DOI:** 10.1371/journal.pbio.1000579

**Published:** 2011-01-18

**Authors:** Naomi R. Wray, Shaun M. Purcell, Peter M. Visscher

**Affiliations:** 1 Queensland Institute of Medical Research, Brisbane, Australia; 2 Psychiatric and Neurodevelopmental Genetics Unit, Center for Human Genetic Research, Massachusetts General Hospital, Boston, Massachusetts, United States of America; 3 Stanley Center for Psychiatric Research, The Broad Institute of Harvard and MIT, Cambridge, Massachusetts, United States of America; The Wellcome Trust Centre for Human Genetics, University of Oxford, United Kingdom

## Introduction

Complex traits and diseases, such as body-mass index, height, diabetes, heart disease, and psychiatric disorders are undoubtedly caused by multiple genetic and environmental factors, although it has been a major challenge to identify specific genes. Recently, genome-wide association studies (GWAS) have resulted in the detection of many robustly associated single nucleotide polymorphism (SNP) variants across a range of outcomes [1], although for any particular disease or trait the SNP variants detected explain only a fraction of the total genetic variance calculated from family studies. The gap between the two has been termed the “missing heritability” [2],[3]. Many reasons for the missing heritability have been given [3]. One plausible explanation is that rare variants, which existing GWAS platforms are not designed to capture, make significant contributions to the heritability of many traits and diseases. It is indeed likely that many multifactorial and heterogeneous phenotypes will be influenced by a diverse array of genetic factors that span the spectrum from private mutation to common variant. Dickson and colleagues [4],[5] recently took a step further, by arguing that rare variants might explain not only some of the heritability that is currently missing, but also that they may be the cause of a proportion of detected associations between complex traits and common SNPs from GWAS. Based on computer simulations, they proposed that some constellations of variants within a narrow frequency and effect size range can account for “many” of the observed associations between complex traits and common SNPs from GWAS. This is a strong claim and one that they say has important implications for the “design of future studies to detect causal variants.” It is of great importance to the research community to establish whether “many” represents an important proportion of GWAS results to date, since indeed this can impact on decisions of experimental design and allocation of research funds.

**Figure 1:**
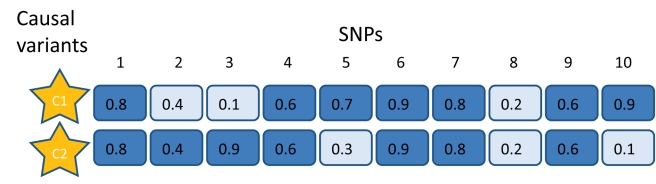
LD between causal and genotyped SNPs and synthetic association. SNPs 1–10 are independent SNPs in a short chromosomal region, with population frequencies indicated by the values in the box. Rare mutations tend to be younger than common mutations. A mutation event in the region creates causal variant C1. C1 has a higher probability of arising on the major allele (dark) of any SNP than the minor allele (light). However, in the absence of recombination, the highest associated SNP will be the one where C1 is coupled (see Box 2) with the SNP allele of lowest frequency, SNP 3; recombination between the SNP and the causal variant could break down this synthetic association. An independent mutation event in the region gives rise to a second causal SNP, C2. Again C2 has higher probability of arising on the major allele of each SNP. If C2 had been the only mutation in the region then SNP 10 would be the most highly associated, as the coupled allele has lowest frequency. However, when both events arise in the same region, the associations at SNPs 3 and 10 are partially masked as they carry risk variants on both their alleles. C1 and C2 arise on the same background allele for many SNPs, but SNP 8 has the allele of lowest frequency that harbours both risk alleles. In the absence of recombination, and depending on effect size, the highest association might be with SNP 8, rather than SNPs 3 or 10. Individuals are very unlikely to carry both C1 and C2. As more causal variants arise in the region, the most associated SNP will be the one with a detectable difference in the contribution to risk from the risk alleles harboured on each allele. Other representations of synthetic association could be viewed in parallel with this representation [4],[5],[16].

Dickson et al. define *synthetic association* as the association of a genotyped common marker resulting from multiple unobserved low-frequency causal variants (see Figure 1). The variance contributed by the causal variants would be much higher than variance explained by the associated genotyped SNP, because the genotyped SNPs will not “tag” (see Box 1) the causal variants with great precision, thus leading to the “missing” heritability from GWAS. Importantly, synthetic associations may arise many hundreds of kilobases (kb) from the site of the causal variant(s), which would hamper attempts to locate the causal variants responsible for association signals by fine-mapping. Dickson et al. claim that rare variants can give rise to synthetic associations that are similar to many observed GWAS associations. As we show below, however, synthetic associations in fact tend to differ in some important ways to observations from GWAS. Furthermore, even if rare variants can, in principle, give rise to associations detectable in GWAS, the converse proposition (that, for a given trait, many, or even any, detected GWAS associations arise from rare variants) does not automatically follow.

**Box 1. The Dickson et al. Genetic Model and Simulations**

Dickson et al. [4] used coalescence theory (Box 2) [19] to simulate patterns of LD that are consistent with an evolutionary process, and then mimicked a GWAS by simulating cases and controls and performing association with disease status and common tagging SNPs (MAF>0.05). Specifically, each simulation was of a genomic region of length 100 kb (representing on 1/30,000^th^ of the genome). To generate realistic patterns of SNP frequencies they assumed an effective population size of 10,000 and a mutation rate of 10^−8^. Within a 100 kb region up to 9 causal SNPs, each with frequency between 0.005 and 0.02 were allocated to influence disease (causal SNPs). Therefore, at a locus with 9 such variants, ∼20% of the general population would be expected to carry at least one disease risk allele. The baseline probability of disease was 1% or 10%, and each risk variant had the same increased risk for disease (genotype relative risk, GRR, see Box 3) compared to the baseline. Each simulation generated 10,000 haplotypes of the 100 kb region. Individuals in the population were simulated by sampling, with replacement, pairs of haplotypes; these were allocated case or control status based on the probability of disease associated with the number or risk loci they carried (with GRR combining multiplicatively when an individual carried multiple risk alleles—this is not a common event, only about 1% of individuals will carry more than one risk allele when there are 9 causal SNPs in the 100 kb region). A case control study was simulated by selecting equal numbers of cases and controls. The simulations varied three parameters – the number of causal SNPs (1,3,5,7,9), the sample size of the case control study (2,000, 4,000, 6,000) and the GRR associated with each risk allele (2,3,4,5,6). Most simulations were conducted in the absence of recombination. The more realistic scenario of recombination (comparing different rates) was considered only when GRR = 4. The simulation of recombination divided the 100 kb region into 200 fragments of 500 bp with no recombination within, and only recombination between, segments. Additional simulations also considered 9 causal variants of GRR = 4 in a 10 Mb region and recombination of 1 cM/Mb.

**Box 2. Glossary of Linkage Disequilibrium**

We consider two loci on a chromosome. The causal locus has alleles C and c and the genotyped marker (SNP) has alleles M and m. These alleles have frequencies *p_C_, 1−p_C_, p_M_,1−p_M_*. The loci can make four possible haplotypes CM, cM, Cm, cm with frequencies *p_CM_, p_Cg_, p_cM_, p_cm_*

**Linkage Equilibrium** – When the frequencies of haplotypes are the frequencies expected from the random association of the alleles , e.g., *p_CM_ = p_C_ p_M_*

**Linkage disequilbrium (LD)** – The non-random association between alleles on a chromosome, e.g., *p_CM_ >p_C_ p_M_*. Recombination breaks down linkage disequilbrium.

**Recombination** – Chromosomal cross-over between the paired chromosomes during meiosis so that the chromosomes passed to offspring comprise a mixture of the chromosomes inherited from its two parents. If the cross-over event occurs between loci C and M, then the LD between them is broken down in the transmitted chromosome. It may take several generations or multiple recombination events to have a substantial impact on the LD in the population.

**Coupled alleles** – Alleles at two loci that tend to be found together on a chromosome. For example, a locus with one rare allele (rare allele C, common allele c), will usually only make three chromosomal haplotypes with any other locus (Minor allele M, major allele m): CM, cM,cm. In this example, the rare allele C is only found in the population coupled with the allele M. This is called *complete LD*. Recombination breaks down the coupling of alleles, so that all four haplotypes exist in the population. However, while there is linkage disequilibrium the coupled alleles are those making combinations of haplotypes with frequency greater than expected if there was linkage equilibrium.

**Measures of LD** –The two commonly used measures of LD are *r* ^2^ and |D'|, both scale the covariance between the loci, *D* = *p_CM_−p_C_ p_G_*, but in different ways. *r* ^2^ = *D^2^*/(*p_C_ p_M_* (1−*p_C_*)(1−*p_M_*)), so *r* is the correlation between the loci, which scales *D* by the standard deviation of allelic frequency at the two loci. When *p_C_* < *p_M_* and C and M are coupled and |*D*'| =  *D*/*p_C_*(1-*p_M_*), so that *D* is scaled by the maximum allelic association possible given the allele frequencies at the two loci. Rare variants often make only three haplotypes with common SNPs, in this case *r* ^2^ can be close to zero while |*D*'| = 1.

**Perfect LD** – When the alleles at one locus (C and c) have the same frequency as the alleles at another locus (M and m) and when the alleles are perfectly coupled so that only two haplotypes exist CM and cm. In this case *r* ^2^ = |*D*'| = 1.

**Complete LD** – When the alleles at one locus (C and c) have different frequency from the alleles at another locus (M and m), but alleles from the C and M locus are coupled as much as is possible given the different alleles frequencies. In this case, only three haplotypes exist in the population e.g., CM,cM,cm. In this case |D'| =  1 and *r* ^2^ can range from very close to zero to 1 (when *r* ^2^ = 1, the allele frequencies of the two loci are equal and there is perfect LD). The value of *r* ^2^ depends on the allele frequency difference between the two loci.

**Maximum** ***r*** **^2^** – The maximum *r* ^2^ possible between two loci given their allele frequencies occurs when the two loci make only three haplotypes so that there is complete LD. If C has the lowest frequency out of C, c, M and m and if allele C is coupled with allele M where M might be either the minor or major allele at this locus then the difference in allele frequencies between the couple loci is *v*  =  *p_M_ −p_C_.* The maximum *r* ^2^ between them is 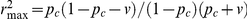 [20]. If allele C is very rare then 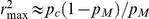, and when *p_M_* is close to 0.5, 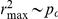.

**Tagging** – When a genotyped SNP that is in LD with a non-genotyped variant, the genotyped SNP tags the non-genotyped variant.

**Coalescence theory** – A population genetics model of inheritance relationships among alleles at a given locus. The coalescence of two alleles is the most recent point (going back in time) at which they shared a common ancestor. Simulation under coalescence theory is an efficient way to generate a realistic distribution of SNP frequencies and LD between them.

**Figure 2:**
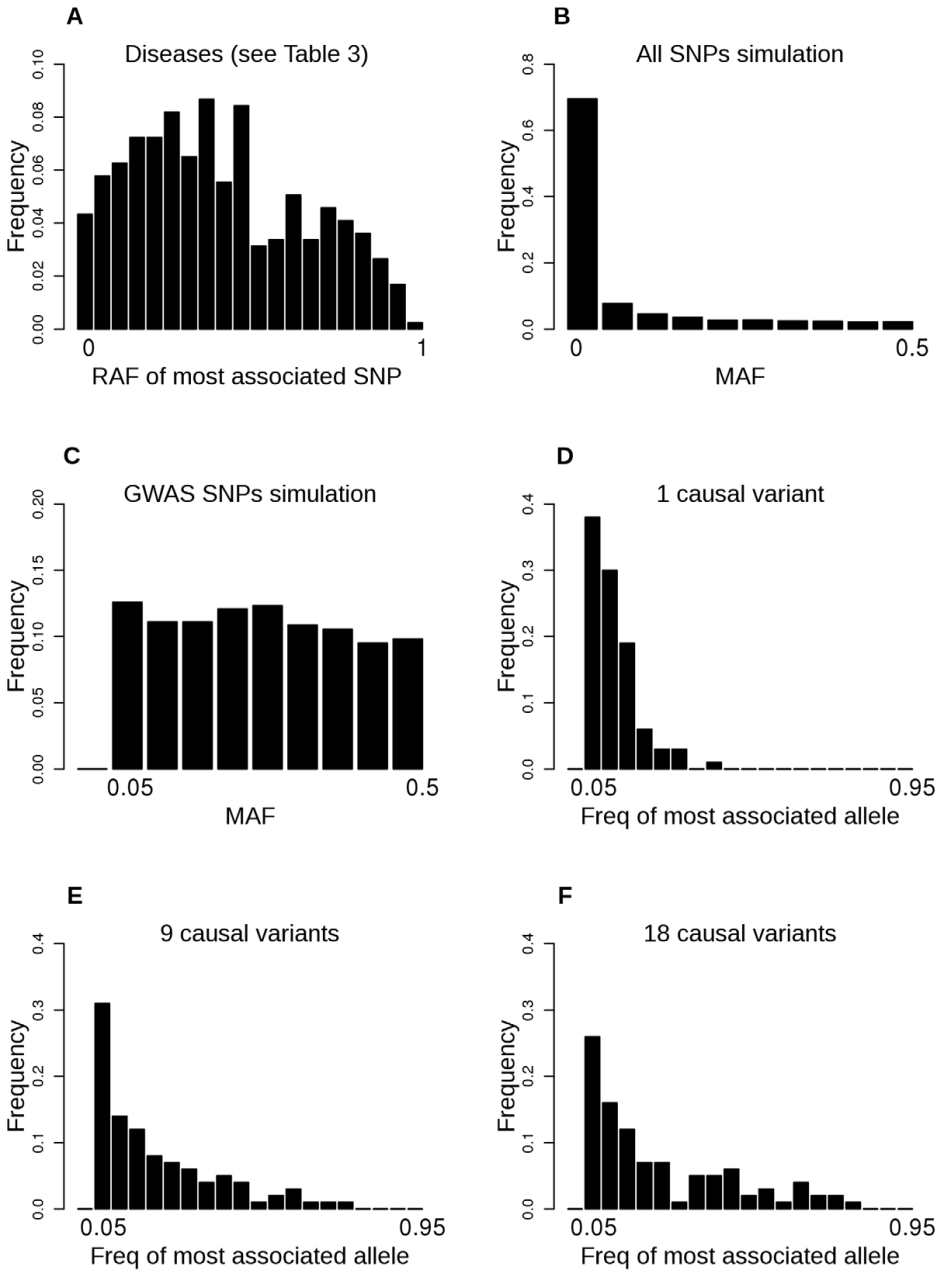
Frequency distributions of a) the risk allele frequency of the most associated SNPs listed in the GWAS Catalog [1] for the diseases in Table 3. b) MAF of all SNPs simulated under the coalescence model, c) MAF of SNPs used in analyses to be representative of SNPs included in GWAS. d–f) Coupled allele of most associated SNP from simulations of 1, 9, or 36 causal variants in a 100 kb region.

The study of Dickson et al. [4] is the first to consider, in detail, a genetic architecture of multiple rare variants within the framework of GWAS analyses. For ease of discussion, we use the terms rare, common, and very common alleles, but the cut-offs between them is necessarily somewhat arbitrary. For the purposes of simulation, Dickson et al. define rare variants as having risk allele frequency (RAF) 0.005–0.02 and define common SNPs to be representative of those used in GWAS studies (minor allele frequency, MAF>0.05). An important proportion of GWAS associations have risk alleles in the very common frequency spectrum (RAF>0.3) (Figure 2a). We will show that it is unlikely that such associations are driven by synthetic associations with single or multiple rare causal variants. We set out to understand and clarify their model and its implications in order to answer three questions:

- What is the expected frequency distribution of the most associated genotyped SNP under the Dickson et al. model?
- How many loci explain total genetic variance of complex disease under the Dickson et al. model?
- Using results from the GWAS of the International Schizophrenia Consortium as an example, are the results of Dickson et al. supported by empirical observation?

## What Is the Frequency Distribution of the Most Associated Genotyped SNP under the Dickson et al. Model?

The simulations of Dickson et al. (Box 1) show that for the genetic models they consider, SNPs with frequency typical of those represented on GWAS panels could tag single or multiple causal variants. They show the likely frequency of the most associated genotyped SNP (their Figure 5), which provides a benchmark for comparisons with empirical data. To understand the results of Dickson et al requires an understanding of linkage disequilibrium (Box 2) between rare and common variants. We first consider the situation of a synthetic association resulting from a *single* causal variant. In association studies, the genotyped SNP is unlikely to be the causal variant, but how likely is it that a GWAS reported association, the most associated common SNP in a region, tags rare causal variant(s)?

**Figure 3:**
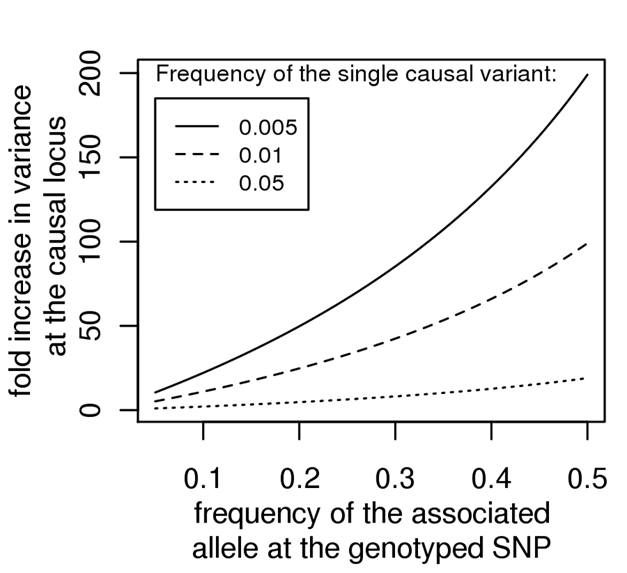
Minimum fold increase in genetic variance at single rare causal locus given the frequency of the risk allele at the genotyped associated locus. The minimum fold increase is calculated as 1/*r* ^2^, with *r* ^2^ calculated as the maximum *r* ^2^ given the frequency of the trait increasing allele at the genotyped SNP and the frequency of the causal allele (see Box 2).

In Figure 3 we show the minimum fold increase in genetic variance at a *single* causal locus compared to the genetic variance explained at a genotyped locus with a given RAF, for causal variants with allele frequencies of 0.005–0.05; the relationship is 1/*r* ^2^ (where *r* ^2^ is a measure of LD between the variants, see Box 2). The *maximum r* ^2^ (see Box 2) between a rare causal variant and SNPs typically included on GWAS chips is very low (Table 1). For example, when the frequency of an associated SNP allele is in the range of 0.2 to 0.5, the variation *contributed* by a causal variant of frequency 0.01 is at least 25 to 100 times larger than that detected at the genotyped SNP. In case-control studies, we can calculate the odds ratio at the causal variant that would be needed to generate the odds ratio detected at the genotyped SNP, which depends on the allele frequencies at the two loci (see Box 3). When the frequency of an associated SNP allele is in the range of 0.2 to 0.5, a detected OR of 1.1 implies that a causal variant of frequency 0.01 must have OR 3 to 6 and a detected OR of 1.3 implies an OR at a causal locus of 4 to 16 (Table 2). As noted by Dickson et al., such effect sizes would be detectable in linkage studies of large pedigrees, or of multiple smaller pedigrees if multiple rare variants occur at the same chromosomal locus and so presumably would have been found already. Therefore, if each GWAS associated SNP represented a synthetic association with a *single* causal variant then, for some traits, we would have already explained the heritability several times over. But what about synthetic associations caused by *multiple* rare variants?

**Table 1:** Maximum *r* ^2^ possible between a rare causal variant and a genotyped SNP, which occurs when the rare causal variant is coupled with the minor allele of the genotyped SNP (see Box 2).

Freq of causal variant (*p_C_*)	Freq of genotyped SNP (*p_M_*)					
	0.05	0.10	0.20	0.30	0.40	0.50
0.005	0.10	0.05	0.02	0.01	0.01	0.01
0.01	0.19	0.09	0.04	0.02	0.02	0.01
0.02	0.39	0.18	0.08	0.05	0.03	0.02

The frequencies of the causal variants listed reflect the minimum, approximate mean and maximum considered by Dickson et al.

**Table 2:** Expected odds ratio at a single causal variant given observed odds ratio of 1.1 at the genotyped SNP (see Box 3).

Freq of causal variant (*p_C_*)	Freq of genotyped SNP (*p_M_*)					
	0.05	0.10	0.20	0.30	0.40	0.50
0.005	2.0	3.0	5.0	7.0	9.0	11.0
0.01	1.5	2.0	3.0	4.0	5.0	6.0
0.02	1.3	1.5	2.0	2.5	3.0	3.5

The frequencies of the causal variants listed reflect the minimum, approximate mean and maximum considered by Dickson et al.

**Box 3. Glossary of Terms Underlying Variance Explained by a Locus on the Liability Scale**

We assume a single locus with two alleles, the non-risk allele, c and the risk allele, C. The frequency of the risk allele is *p*, so that the frequency of the genotypes cc, cC and CC in the population are (1−*p*)^2^, 2*p*(1−*p*) and *p* ^2^, assuming Hardy-Weinberg equilibrium.

**Genotype relative risk (GRR):**

GRR expresses the increased risk of disease associated with a single risk allele and is represented by the single character *γ*, so that under a multiplicative model of disease, the probability of disease for the three different genotypes are P(D|cc) = ϕ, P(D|Cc) = ϕ*γ* and P(D|CC) = ϕγ^2^. If the disease prevalence in the population is, *K* and P(D) = *K* = (1−*p*)^2^(D|cc)+2*p*(1−*p*)P(D|Cc)+*p* ^2^P(D|CC), then ϕ  =  *K*/(1+*p*(γ−1))^2^. For high GRR ϕ γ^2^>1, in this case P(D|CC) should be constrained to 1, and then ϕ = *K*/(1+*p*(γ−1))^2^_._ Dickson et al. chose to allow disease prevalence to vary, by fixing ϕ as a defined baseline probability.

**Odds Ratio (OR):**

The OR for heterozygotes compared to homozygotes of the non-risk allele is a function of the ratios of the probabilities of disease and not disease (ND) for the different genotype classes (P(D|Cc)/P(ND|Cc))/(P(D|cc)/P(ND|cc)) = γ(1−ϕ)/(1−ϕγ).

**Equivalence of GRR and OR:**

As *K*→0, OR→*γ*. Since *K* is small for most complex genetic diseases, GRR for heterozygotes and OR are used interchangeably. OR can be estimated from data as it is robust to the inflated P(D) in case control studies—i.e., where the frequency of cases is often ∼0.5, rather than *K*.

**Variance explained on the liability scale:**

If the associated variant has effect size GRR and allele frequency *p*, then the genetic variance in liability explained by the variant can be calculated from the mean liability associated with each genotype class (Table 4), but can be approximated as *V_G_* = 2*p*(1−*p*)ln(OR)^2^/*i* ^2^, where *i* is the mean liability (expressed in standard deviation units) of the diseased group calculated from normal distribution theory assuming a disease prevalence, *K*. *i* = *z*/*p*, where *z* the height of the standard normal curve at the liability (*T*) that truncates the proportion *K* on the standard normal curve. The residual variance is assumed to be normally distributed with variance 1, so the variance explained by the locus on the liability scale is *h* ^2^ = *V_G_*/(1+*V_G_*). The assumptions of normality used in the liability threshold model break down when each rare locus contributes a large proportion of the variance.

**Variance explained at causal versus marker loci:**

If the variance in disease liability explained by a causal locus is *V_C_*, then the variance explained at the genotyped locus is *V_M_* = *r^2^V_C_* (where *r^2^*is the linkage disequilibrium described in Box 2). Therefore, if we estimate the variance explained by a common genotyped genetic marker, *V_M_* then we can estimate the variance explained by the causal variant is expected to be *V_C_* = *V_M_/r^2^*. This relationship holds for quantitative traits but breaks down for disease traits when *V_M_*−*V_C_* is large and so cannot be used for calculating the variance explained by the causal variant. Instead we calculate the odds ratio at the causal locus and calculate the variance explained from that.

**OR at the causal locus given the estimate of the OR at the genotyped SNP:**

The OR at the causal locus OR_C_ can be calculated as a function of the OR at the genotyped locus, OR_C_ = 1+(*OR*−1)*p_M_*/*p_C_* [21].

Dickson et al. [4] consider up to nine rare causal variants within a 100 kb genomic region. We repeated their coalescent simulations (see Text S1), but using a quantitative trait for simplicity, although the same principles apply for disease outcomes. For a quantitative trait the simulated 100 kb haplotypes have quantitative values which are a function of the number of causal variants they carry, since we assume (like Dickson et al.) that all causal SNPs have the same effect size. We allowed for recombination at the standard rate of 1 cM/Mb across the whole 100 kb region and varied the number (*k*) of rare causal SNPs between 1 and 18. We investigated the frequency distribution of the most associated SNP allele. To make this representative of SNPs included in GWAS studies, we retained all SNPs with MAF>0.2 and a proportion of SNPs of lower MAF to generate an approximately uniform distribution of genotyped SNPs (Figure 2b–c). As the number of causal SNPs increased we found that the frequency of the most associated common variant changed, based on the most likely coupling pattern. When *k* = 1, as discussed above, out of all common SNPs segregating in the population, the genotyped SNP which tags the most variance from the causal SNP (i.e., has the highest *r* ^2^) is the SNP with the lowest MAF in which the minor allele is coupled with the rare causal variant. When *k*>1, the genotyped SNP which explains the highest proportion of variance (*R* ^2^) of the quantitative trait is a SNP where one of its alleles is coupled with more rare variants than the other allele. The highest *R* ^2^ across all SNPs segregating in the population will occur for the SNP with the lowest allele frequency that fulfils this criterion. From the simulations, the mean frequency of the most associated genotyped SNP allele was 0.13 for one rare variant and <0.3 for up to 18 rare variants with a trend that the frequency of the most associated allele increased with more rare variants in the region. This is consistent with a slight trend towards the observed lower *R* ^2^ between composite value of the *k* causal variants and the common SNP allele for increasing *k*, implying a lower power of detection of synthetic associations with higher RAF. Histograms of the frequencies of the allele coupled with the most number of risk variants of SNP that generates the highest *R* ^2^ for *k* = 1, 9, and 18 (Figure 2d–f); these distributions are quite different from that observed from GWAS results (Figure 2a). Of course, if a region harbours 9 rare variants of frequency 0.01, about 20% of the population are expected to carry at least one of them. If the true genetic architecture of a region was one of many rare variants, then the effect sizes necessarily attributable to each of them must be small, otherwise the region would have been unambiguously identified in linkage studies and would explain a large proportion of genetic variation in the population.

From our simulations, we also made some important novel observations. We found that the total variance in haplotypes for *k* causal variants was approximately *k* times the variance explained by 1 causal variant, implying that within a 100 kb block with mutations arising randomly and allowing for recombination at the usual rate of 1cM/Mb that the causal variants were in approximate linkage equilibrium. We found that the mean *R* ^2^ between haplotype values and the presence/absence of a genotyped SNP allele for the most associated genotyped SNP was ∼0.09 for 1 rare causal variant and ∼0.08 for 9 rare variants or more. In other words, for this particular model, the proportion of variance attributable to the causal variants explained by the most associated genotyped SNP was insensitive to the number of rare variants simulated, so that on average the most associated SNP explains less than 10% of the genetic variation contributed by the locus. Furthermore, we found that compared to this average, very common associated SNPs explained a smaller proportion of the total variance than less common variants, implying that the variance explained by the causal variants would have to be very high for such very common alleles to have been detected given the power of typical GWAS to date. As noted by Dickson et al., as sample sizes increase the power to detect variants including synthetic associations increases, but in all cases we would expect to see the distribution of RAF skewed towards less common variants.

Since the distribution of the frequency of the most associated allele observed from GWAS is not consistent with an important contribution of multiple rare variants and since the variance attributable to the causal locus would have to be unrealistically high to be detectable by GWAS conducted to date, we conclude that multiple rare variants are unlikely to explain an important proportion of GWAS results, particularly for associations with very common alleles. GWAS of larger sample size will undoubtedly identify more associations and will point to additional regions in the genome for follow-up studies. A recent GWAS of height of >180,000 individuals [6] has identified 180 loci enriched for genes that are connected in biological pathways and that underlie skeletal growth defects. Much larger sample sizes are needed for GWAS of disease than have been currently conducted to achieve the same power afforded to the height study (e.g., ∼50,000 cases and 50,000 controls for schizophrenia [7]).

## How Many Loci Explain Total Genetic Variance of Complex Disease under the Dickson et al. Model?

The simulations of Dickson et al. (Box 1) are parameterised in terms of the GRR (genotype relative risk, Box 3) of the causal variants. They consider a range of effect sizes (GRR 2–6) for each of the causal variants and GWAS case-control samples of 2,000 to 6,000. Although such simulations allow evaluation of the ability to detect synthetic associations and allow extrapolation to smaller effect sizes and larger sample sizes, the results generated from the selected parameters do have an impact on the perception of the importance of synthetic associations. To interpret the genetic architecture of their model we calculated the variance explained under the liability threshold model (see Box 3), assuming that each causal variant has frequency 0.01 (the average of the range considered, which will make our results conservative since the distribution of variance explained for causal variants of frequency 0.005–0.02 is not symmetric). When baseline disease probability was 0.01, a single GRR of 2 corresponds to a variant explaining 0.15% of the variance in liability and a GRR of 6 corresponds to 1.2% of the variance in liability. For 9 causal variants the variance explained is approximately 9 times the value for a single locus, i.e., 1.4% for GRR = 2 and 13% for GRR = 6. Table 4 shows how the probability of disease in the population increases under the Dickson et al. model, which fixes the baseline (no risk alleles) probability of disease; for the model with baseline probability of disease of 10% for 9 causal variants each of GRR = 6, the actual probability of disease in the population is 19%. For more natural benchmarking, we have undertaken the calculations fixing the probability of disease in the population. Assuming a disease with a heritability of 0.8, we calculate the maximum number of these 100 kb loci possible in the genome if all the genetic liability variance were attributable to loci like this, as this provides an upper limit to the contribution of such loci. The maximum number of loci can be high (maximum 543, with 543 100 kb blocks representing <2% of the genome) when there is only a *single* causal variant of GRR = 2 within a 100 kb region; we showed in the previous section that very common genotyped SNPs are unlikely to ever be the most associated SNP from a GWAS tagging single rare causal variants. When there are 9 causal variants, the maximum number of 100 kb regions required to explain all the variance for the models considered by Dickson et al. is only 59 (<0.2% of the genome), occurring when the GRR of each causal variant is 2 so that each block explains 1.4% of the variance. Dickson et al. showed that if 9 causal variants were segregating in a 10 Mb block that the most associated SNP may be positioned several Mb from the most distal causal variant it is coupled with. Even if associations from causal variants spanned several Mb for synthetic associations, to have an important contribution to disease variance we would expect to see associations concentrated in only a fraction of the genome. This will be a testable hypothesis as sample sizes increase; results to date do not support such a concentration of associations. Since the simulation parameters used by Dickson et al. generate a relatively small number of rather large loci, their results may generate a false sense of the relative importance of the contribution of synthetic associations to GWAS results observed to date.

## Using Results from the GWAS of the International Schizophrenia Consortium as an Example, Are the Results of Dickson et al. Supported by Empirical Observation?

In 2009, the International Schizophrenia Consortium (ISC) reported an analysis that supported a genetic model for schizophrenia that included a substantial number of common variants of small effect. Dickson et al. claimed, of the ISC analysis, that multiple rare variants in a region are capable of acting over large distances to create associations in common variants similar to those observed by the ISC. However, as we have shown above, a genetic architecture dominated by rare causal variants leads to a distribution of risk alleles of detected associations skewed to low frequencies, which is not consistent with the ISC data, as we show below.

**Figure 4:**
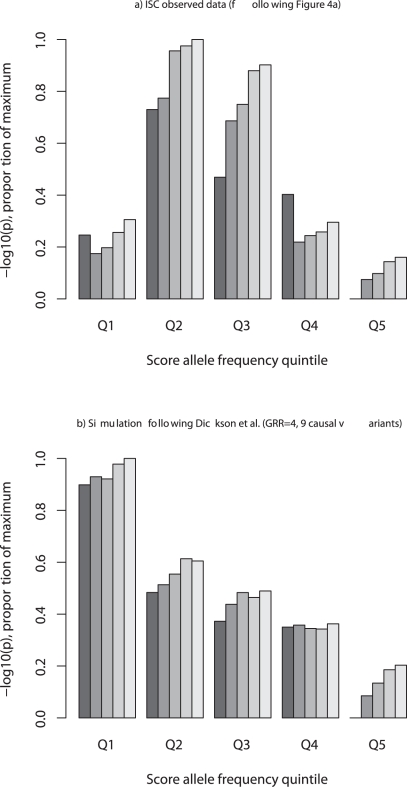
Polygenic analyses following the International Schizophrenia Consortium [12]. a) The original results for polygenic score analysis in the ISC, when stratified by quintile of risk-increasing allele frequency (Q1 being the lowest risk-increasing allele frequency, Q5 the most common; the range is between 0.02 and 0.98). b) We repeated these analyses on simulated data, generated under a “rare variant only” model and using the same simulation procedure as Dickson et al., assuming that risk loci harbor 9 causal variants, GRR = 4, MAF 0.005–0.02). The pile-up of signal in the lower quintiles, which is expected under Dickson et al.'s model, is clearly not consistent with the observed ISC results. In the simulations, SNPs are generated through a coalescent process; a subset of SNPs is selected as “genotyped” to represent the marker density, frequency distribution and LD profile observed in the original ISC study (which has properties that are typical of most GWAS, including the under-representation of low frequency variants). The y axis is the –log_10_P from the logistic regression of case-control status on profile score in an independent “target” case-control sample using a score calculated as the number of alleles identified as associated (with p-value less than a threshold p_T_) in the discovery case-control sample association analysis, scaled within each figure as so that the maximum value observed for five significance thresholds (p_T_ = 0.1, 0.2, 0.3, 0.4, and 0.5, plotted left to right in each quintile) is scaled to 1 and the minimum is scaled to zero.

Schizophrenia is a common complex disease for which several studies [8],[9],[10],[11] (including one by the ISC) have identified a role for very rare structural variants in its underlying genetic architecture. In a GWAS comprising of 3,322 cases and 3,587 controls [12], the ISC analysis presented evidence that some common genetic variants also contribute to the genetic architecture, demonstrated by the highly significant signal of association of in an independent case-control sample based on a profile of the top 50% of associated SNPs from the ISC study. In the ISC study we undertook simulations of a wide range of genetic architectures in which we varied allele frequency, effect size distributions and the extent of LD between causal and genotyped SNPs. Although we could reject many of the simulated genetic architectures as not being representative of the observed results, many others were consistent with our results, but all consistent models included common variants as well as rare causal variants. Dickson et al. cites the ISC paper and specifically implied that multiple rare variants could explain the ISC results. Since our only conclusion was that the genetic architecture must include common variants (we specifically stated “our results do not exclude important contributions of rare variants for schizophrenia”), their statement must be interpreted that they believe a model without common variants could explain the ISC results. Within the ISC GWAS analysis we specifically investigated whether a rare variants-only model could fit the observed distribution of the frequencies of associated variants. Those results corroborate the results from the coalescent simulations we have undertaken here, namely that a rare variants-only model predicts a skewed distribution of associations for risk alleles of low frequencies. While, as noted in the ISC paper, we did observe an enrichment of associations with lower-frequency common alleles, we did not observe the substantial excess predicted by a rare variants-only model. Using exactly the same coalescent simulation models and methods as Dickson et al., we repeated the polygenic analysis presented by the ISC. For comparability with the ISC analyses, we sampled variants from the full simulated set to obtain a MAF distribution similar to that observed in the ISC GWAS, and we further restricted analysis to an LD pruned set (no pairwise LD *r* ^2^>0.25). Using the same discovery/sample framework described in [12], we stratified variants into quintiles according to the frequency of the risk-increasing allele. In contrast to the results for the observed ISC data (Figure 4a, following Figure 4a of [12]), the results from simulations under Dickson et al.'s model (Figure 4b) show a marked skewing towards the lower quintiles, indicating that lower frequency SNPs on the GWAS platforms do a better job at tagging rare variants than more common SNPs. Therefore, the empirical results from the ISC GWAS are not consistent with the model presented by Dickson et al. being a general explanation of common variant association.

In our simulations, as in those of Dickson et al., we observed genome-wide significant results a good proportion of the time (90% for loci with 9 causal variants with GRR = 4), suggesting that if variants of this effect size and frequency ∼0.01 exist (the benchmark simulations of Dickson et al.) then they would have been detected by standard single SNP analysis in the ISC GWAS. It is important to note that this ability is achieved using standard GWAS chips, which are designed to have a biased distribution of allele frequencies (higher proportion of common variants) relative to the distribution of SNP allele frequencies in the population (and the distribution of SNP frequencies generated by the coalescent simulations).

## Conclusion

Under evolutionary theory we expect a genetic architecture of many more rare than common variants and a negative correlation between effect size and MAF [13],[14]. Lessons learned from Mendelian disorders lead us to expect that genes involved in the genetic architecture of disease will harbour many causal variants [15]. In association studies it is well recognised that genotyped SNPs tag other, unobserved, variants in the genome and that when a SNP is identified as associated it is unlikely that the SNP itself is the causal variant. Whether synthetic associations with rare causal variants represent a significant proportion of associations detected in GWAS depends on the true, but mostly unknown, genetic architecture.

Dickson et al. [4] used simulation to determine if associations detected in GWAS could reflect synthetic associations of single or multiple rare causal variants. Their abstract states, “We show that they are not only possible, but inevitable, and that under simple but reasonable genetic models, they are likely to account for or contribute to many of the recently identified signals reported in genome-wide association studies.” Their results have been interpreted by the scientific community to imply that this mechanism could apply to an important proportion of associations detected in GWAS. Benchmarking the importance of synthetic associations with rare causal variants is relevant for determining the direction of future research.

The relevance of the simulations conducted by Dickson et al. depends on whether the genetic architecture they assumed is a realistic representation of nature. Empirical observations do not generate the pattern of results generated by their genetic model, which implies that their model applied genome-wide does not reflect the typical architecture of complex disease. If their model of genetic architecture is only representative of a small proportion of the genome and represents only a small proportion of the genetic variance, their results cannot be representative of most GWAS results. A genetic architecture of rare variants only will generate a distribution of associated variants very heavily skewed towards genotyped SNPs with low RAF; this does not agree with observation. Multiple rare variants within short genomic regions could generate associations with alleles of higher frequency including major alleles, but the variance explained by the causal variants would need to be so high that firstly, it should have been detected by linkage studies and, secondly, it would imply very few such loci across the genome for the mechanism to be considered important. Results from GWAS suggest that associations are not concentrated in a handful of locations and show that high frequency alleles have been reported for most diseases (Table 3).

**Table 3:** Associations of SNPs with very common SNP alleles detected in GWAS associations downloaded from the GWAS Catalog [1].

Disease	No. SNPs with associations^a^	Proportion of associationsFor RAF > 0.3	Proportion of associationsFor RAF > 0.8
Asthma	11	0.73	0.18
Ankylosing Spondylitis	8	0.88	0.13
Age Related Macular Degeneration	11	0.64	0.09
Bipolar Disorder	42	0.57	0.07
Breast Cancer	19	0.53	0.05
Celiac Disease	40	0.60	0.08
Chronic Kidney Disease	28	0.54	0.00
Coronary Heart Disease	13	0.69	0.15
Crohn's Disease	37	0.64	0.03
Multiple Sclerosis	33	0.64	0.09
Pancreatic Cancer	19	0.68	0.05
Parkinson's Disease	10	0.80	0.40
Prostate Cancer	27	0.62	0.07
Type 1 Diabetes	41	0.73	0.10
Type 2 Diabetes	28	0.64	0.11
Schizophrenia	17	0.47	0.12
Systemic lupus erythematosus	29	0.31	0.03

SNPs are included in the catalog for GWAS of >100,000 SNPs, SNP p-value <10^−5^ in the overall (initial GWAS + replication) population or SNP p-value <10^−5^ when there is no replication stage. To avoid duplications, if the chromosomal cytogenetic band region contained >1 associated SNP, the one with the lowest RAF was selected.

**Table 4:** Estimating the maximum number of 100 kb regions containing either 1 or 9 causal variants of frequency 0.01 possible in the genome.

	Probability of disease benchmark 1%						Probability of disease benchmark 10%					
	1 causal SNP of frequency 0.01 in 100 kb region			9 causal SNPs^a^ of frequency 0.01 in in 100 kb region			1 causal SNP of frequency 0.01 in 100 kb region			9 causal SNPs^a^ of frequency 0.01 in in 100 kb region		
GRR	% phenotypic variance explained^b^	Max number Loci^c^	Population probability of disease	% phenotypic variance explained	Max number Loci^b^	Population probability of disease	% phenotypic variance explained	Max number Loci^a^	Population probability of disease	% phenotypic variance explained	Max number Loci^b^	Population probability of disease
Probability of disease fixed for those carrying no risk alleles, the probability of disease increases with GRR and number of causal SNPs.^d^												
2	0.15	543	0.010	1.4	59	0.012	0.38	208	0.102	3.9	21	0.120
3	0.39	202	0.010	3.7	22	0.014	1.2	69	0.104	15	5	0.141
4	0.66	122	0.011	6.4	13	0.017	2.3	35	0.106	35	2	0.158
5	0.92	87	0.011	11	7	0.020	3.4	24	0.108	37	2	0.173
6	1.2	68	0.011	13	6	0.023	4.6	17	0.110	41	2	0.188
Probability of disease fixed in the population.												
2	0.15	546	0.010	1.3	67	0.010	0.38	211	0.100	3.4	23	0.100
3	0.39	206	0.010	3.3	24	0.010	1.1	71	0.100	11	8	0.100
4	0.64	124	0.010	5.4	15	0.010	2.2	36	0.100	20	4	0.100
5	0.90	89	0.010	7.2	11	0.010	3.2	25	0.100	36	2	0.100
6	1.1	70	0.010	9.0	9	0.010	4.2	19	0.100	38	2	0.100

GRR: Genotype relative risk (see Box 3).

a see Box 3.

b The 9 causal SNPs are assumed to be in linkage equilibrium as found in our coalescent simulations

c Maximum number of loci in the genome assuming a heritability of 80%  = 80/V% , i.e, heritability is 1, this may be an underestimate when GRR is high (see text).

d as in Dickson et al.

Undoubtedly, part of the missing heritability is explained by imperfect LD between the genotyped SNPs and causal variants, including rare causal variants and including multiple rare causal variants concentrated in relatively short genomic regions. Dickson and colleagues [4],[5] give six examples of known synthetic associations detectable in GWAS but generated by rare causal variants, providing compelling evidence for their existence. However, their examples likely represent the high end of effect size of rare causal variants (since in many instances the causal variants had been detected in the pre-GWAS era) and yet, in all examples, the RAF of the most associated tag SNPs (13 were listed across the examples) were less than 0.33. However, the majority of associations detected from GWAS are for very common SNPs (Table 3); although very common associated SNPs are not likely to be the causal variants, they are much more likely to tag causal variants of similar frequency and highly unlikely to represent synthetic associations with single or multiple rare causal variants. Thus, we conclude that synthetic associations created by rare variants are unlikely to explain the majority of GWAS results. That is, because multiple rare variants can create synthetic associations (as nicely shown by Dickson et al.), it doesn't follow that observed associations with common SNPs are caused by multiple rare variants.

Orozco et al. [16], reviewing empirical and theoretical data, and drawing particularly upon evidence from linkage studies, pathway analyses and trans-ethnic studies, also recently concluded that synthetic associations with multiple rare variants cannot be responsible for many reported GWAS associations. The synthetic association hypothesis was also specifically addressed in a recent multi-ethnic study. Waters et al. [17] took 19 variants (15 had RAF>0.3) reproducibly associated to Type 2 Diabetes in Europeans and tested them in 5 racial/ethnic groups (European Americans, African Americans, Latinos, Japanese Americans, and Native Americans). Despite the relatively small sample size for what was essentially a replication study (total size of 14,000 across all groups) the OR for total number of risk alleles in each ethnic group was highly significant, implying ancient causal variants predating the migrations that separated these populations. Waters et al. argued that synthetic associations with rare variants could not explain these common associations with Type 2 diabetes.

Empirical observation suggests that much of the missing heritability is contributed by causal variants (including loci comprising multiple rare variants) having effect size too small to be detected with stringent statistical significance [6],[12],[18]. Larger samples for GWAS are needed to detect these which would directly compete with research funds used in sequencing studies. Our assessment is that that the importance of synthetic associations generated by multiple variants has been overstated. Sample sizes of about 50,000 cases and 50,000 controls are required for a GWAS of schizophrenia [7] to afford the same power in detection of variance explained as a GWAS of 180,000 individuals for height [6]. The height study found that in 13 of 21 loci containing a known skeletal growth gene, the known gene was closest to the most associated variant in the region, leading the authors to make the general conclusion that the likely causal gene is often located near the most strongly associated variant [6]. Genes identified through GWAS harbouring common variants are likely to be good targets for identification of rare variants and for sorting the wheat from the chaff in next generation sequencing studies. We expect that continued GWAS will make valuable contributions to our understanding of many complex traits and will, for some time, remain as one important tool in a growing set of technologies to probe the full spectrum of genetic variation efficiently.

## Supporting Information

Text S1 **Supplementary Information**
(0.03 MB DOC)

## Abbreviations

GRR: genotype relative risk
GWAS: genome-wide association study
ISC: International Schizophrenia Consortium
LD: linkage disequilibrium
MAF: minor allele frequency
OR: odds ratio
RAF: risk allele frequency
SNP: single nucleotide polymorphism
