# Supplementary material for: Synthetic Associations Created by Rare Variants Do Not Explain Most GWAS Results
Source: PLoS Biol. 2011 Jan 18;9(1):e1000579. doi: 10.1371/journal.pbio.1000579 (PMC3022526; doi:10.1371/journal.pbio.1000579)
Supplement: Text S1 — Supplementary Information (0.03 MB DOC) [file pbio.1000579.s001.doc]

**SUPPLEMENTARY INFORMATION**

**Synthetic associations created by rare variants do not explain most GWAS results**

*Naomi R Wray, Shaun M Purcell and Peter M Visscher*

**Evolutionary simulations**

We performed similar simulations to Dickson et al, except we performed coalescent simulation using the program ‘ms’ [1] which allows free recombination across the simulated region under the Fisher-Wright evolutionary model, rather than the approximation to recombination (only between not within fragments) used in the software GENOME. We used the simulations to explore the pattern of LD because the results from the GWAS are a direct consequence of LD between rare and common variants. Specifically, 100 datasets of 10,000 haplotypes of length 100kb were simulated under a neutral evolutionary model. Either a standard recombination rate (1 cM/Mb) or no recombination was used; unless stated otherwise, the results are for the standard rate. We did not simulate a disease phenotype but worked simply with the number of designated causal variants per individual which can be transformed into a quantitative trait by assigning them a value. For each simulation set we selected *k* causal SNPs with the derived allele frequency limited to the range 0.005 and 0.02. Like Dickson et al we considered *k* between 1 and 9, but also tested larger values of *k*. We assigned a value to each causal SNP, with an effect of *a* = 1.0 assigned to the derived allele, which means that the additive value of a haplotype is simply the count of the number of causal variants. Simulations were also run in which the direction of effect of the derived allele was arbitrary (probability of 0.5 for -1 and +1).

For each simulated dataset, we calculated the *R*^2^ values from a regression of the haplotype value (*A*) on the presence or absence of an allele at a common SNP (*x* = 0 or 1). Since *A* is usually 0 or 1, this is essentially a composite LD *R*^2^ measure between a common SNP and multiple rare causal variants. The SD among the *R*^2^ values across all common SNPs with a MAF > 0.05 was calculated and the largest values of *R*^2^ across the common SNPs and the MAF of this SNP were stored. We also calculated the product of var(*A*) and the largest values of *R*^2^ across the common SNPs. This is the genetic variance associated with the common SNP and is proportional to the non-centrality-parameter of a test for association. Statistical power can be calculated from these statistics, without having to simulate phenotypes explicitly.

*Allele frequency and genic variance*

Coalescent simulations generated ~396 SNPs (standard deviation (SD) ~ 28 ) in the 100kb region, ~57 (SD ~ 10) of which have an allele frequency between 0.005 and 0.02, and ~121 (SD ~23) common SNPs with a MAF > 0.05. All these results can be predicted using standard coalescence theory.

The genetic value (*A*) per haplotype was calculated by summing the effect size (*a*) of the causal alleles. We simulated with *a* set to 1 for the derived allele and 0 for the ancestral. In most simulations, *A* is the number of rare variants per haplotype, so the value is almost always 0 or 1. The probability of having 2 rare variants on a single haplotype is approximately 0.01^2^, i.e. low, in models including recombination. The genic variance (var(*A*)) across haplotypes was calculated from all 10,000 simulated haplotypes. The diploid additive genetic variance in the population is simply twice the genic variance because we assume a simple additive model. Approximately, the variance contributed by the 100kb locus is *k**2*p***a*^2^, with *p* the allele frequency of the derived allele and *k* the number of causal variants in the locus. So if the total genetic variance is *h*^2^, then there can be *h*^2^/ (2*p***a*^2^) such rare variants in the genome. If *p* ~ 0.01 and *h*^2^ ~ 1, this implies 50/*a*^2^ loci, with the effect (*a*) measured in phenotypic SD units.

The genic variance was ~ 0.022 for 1 rare variant, and the average allele frequency of the rare causal variants was 0.011. When simulating 9 variants, the genic variance was ~ 0.219, which is only slightly more than 9 times the single locus variance. Therefore, there is little LD between the causal variants. When no recombination was simulated the genic variance was ~ 0.260, which is more than 9x0.022 due to LD between rare variants. From these basic results, other parameters can be scaled. For example, for *a* = 3, the variances increase 9-fold, so 9 times fewer such loci in the genome for a constant genetic variance. When simulating the sign of the effect (i.e., +*a* or –*a*) to be arbitrary (probability 0.5), the contribution of the rare variants to the trait variance was independent again even without recombination. That is, the variance contributed by 9 rare variants is approximately equal to the sum of the variance of each individual variant.

*LD structure*

The mean *R*^2^ between haplotype values *A* and the presence/absence of a common variant for the best common SNP was ~0.12 for 1 rare variant and ~0.10 for 9 rare variants. This value appears insensitive to the number of rare variants simulated: when *k* = 18 was simulated the average was still 0.10. This implies that for this particular model, the best SNP detects, on average, only 10 to 12% of the genetic variation contributed by the locus. The SD or *R*^2^ between common SNPs within a locus was ~0.019 for *k* = 1 and ~0.016 for *k* = 9, so little difference.

In the absence of recombination, when *k* = 1 the common SNP with the highest *r*^2^ will be the SNP with the lowest minor allele frequency that is coupled with the rare variant. When *k* > 1, loci with higher allele frequency can create a higher *R*^2^ if more than one rare variant are coupled with the same allele. For a substantial *R*^2^ to be possible one requirement is that one of the alleles at the locus must be coupled with more rare variants than the other allele. This happens most often with the major allele, but the highest *R*^2^ across SNPs will occur with the allele with the lowest frequency that fulfils this criterion. From the simulations the MAF of the best SNP was 0.10, 0.13, 0.15 and 0.16 for 1, 5, 9 and 18 rare variants, respectively. Hence there is a slight tendency for the best common SNP to have a larger MAF when there are more rare variants. This is consistent with a slight trend towards a lower *R*^2^ between *A* the common SNP allele for increasing numbers of rare SNPs.

For the results presented in the main manuscript we used a distribution of SNP MAF representative of those included in GWAS studies, we retained all SNPs with MAF > 0.2 and a proportion of SNPs of lower MAF to generate an approximately uniform distribution of genotyped SNPs.

***Evolutionary simulations compared to International Schizophrenia Consortium GWAS***

In each of 50 replicates, we simulated from 3 independent regions of 100 kb with 1.5 Mb flanking regions using GENOME [2]with 500 bp fragments, 5×10^-6^ recombination rate (1 cM/Mb) between fragments, and effective population size of 10,000. We simulated 9 causal variants per site with risk allele frequency between 0.005 and 0.02. Each causal variant had a GRR=4. In each replicate, we generated 3000 case/control pairs.

Variants were sampled from simulated variants according to the empirical allele frequencies in the ISC study, restricted to MAF > 0.02 and pruned to obtain a set of variants in approximate linkage equilibrium, using the same parameters as described in the ISC manuscript [3]. Following the ISC, we repeated the polygenic analysis (based on independent discovery and target samples, with 1500 cases and control randomly assigned to each) to estimate the significance of the regression coefficient in the logistic regression of target disease stats on the polygenic score based on variants below discovery sample *p*-values of 0.1, 0.2, 0.3, 0.4, and 0.5 in each of the risk allele frequency quintiles.

REFERENCES

1. Hudson R (2002) Generating samples under a Fisher-Wright neutral model of genetic variation. Bioinformatics 18: 337-338.

2. Liang LM, Zollner S, Abecasis GR (2007) GENOME: a rapid coalescent-based whole genome simulator. Bioinformatics 23: 1565-1567.

3. Purcell SM, Wray NR, Stone JL, Visscher PM, O'Donovan MC, et al. (2009) Common polygenic variation contributes to risk of schizophrenia and bipolar disorder. Nature 460: 748-752.
